# Detection of DNA mismatch repair proteins in fresh human blood lymphocytes - towards a novel method for hereditary non-polyposis colorectal cancer (Lynch syndrome) screening

**DOI:** 10.1186/1756-9966-30-100

**Published:** 2011-10-21

**Authors:** Samar Hassen, Bruce M Boman, Nawab Ali, Marcie Parker, Chandra Somerman, Zohra J Ali-Khan Catts, Akhtar A Ali, Jeremy Z Fields

**Affiliations:** 1 CATX Inc., Gladwyne PA 19035, USA; 2 Graduate Institute of Technology, University of Arkansas at Little Rock, Little Rock, AR 72204, USA; 3 Helen F Graham Cancer Center, Newark DE 19713, USA

**Keywords:** Lynch Syndrome, Hereditary Cancer, MMR proteins, HNPCC, MLH1, MSH2, Lymphocytes, PHA treatment, Western blotting, Cell Culture

## Abstract

**Background:**

A broad population-based assay to detect individuals with Lynch Syndrome (LS) before they develop cancer would save lives and healthcare dollars via cancer prevention. LS is caused by a germline mutation in a DNA mismatch repair (MMR) gene, especially protein truncation-causing mutations involving *MSH2 *or *MLH1*. We showed that immortalized lymphocytes from LS patients have reduced levels of full-length MLH1 or MSH2 proteins. Thus, it may be feasible to identify LS patients in a broad population-based assay by detecting reduced levels of MMR proteins in lymphocytes.

**Methods:**

Accordingly, we determined whether MSH2 and MLH1 proteins can also be detected in fresh lymphocytes. A quantitative western blot assay was developed using two commercially available monoclonal antibodies that we showed are specific for detecting full-length MLH1 or MSH2. To directly determine the ratio of the levels of these MMR proteins, we used both antibodies in a multiplex-type western blot.

**Results:**

MLH1 and MSH2 levels were often not detectable in fresh lymphocytes, but were readily detectable if fresh lymphocytes were first stimulated with PHA. In fresh lymphocytes from normal controls, the MMR ratio was ~1.0. In fresh lymphocytes from patients (N > 50) at elevated risk for LS, there was a bimodal distribution of MMR ratios (range: 0.3-1.0).

**Conclusions:**

Finding that MMR protein levels can be measured in fresh lymphocytes, and given that cells with heterozygote MMR mutations have reduced levels of full-length MMR proteins, suggests that our immunoassay could be advanced to a quantitative test for screening populations at high risk for LS.

## Background

Colorectal cancer is the second most common cause of cancer deaths in western countries including the US. It was responsible for 9% of new cancer cases and 10% of cancer deaths in 2010 in the US [1,2]. Hereditary non-polyposis colorectal cancer (HNPCC), or Lynch Syndrome (LS), is the most common form of hereditary colorectal cancer, accounting for 5-10% of all colon cancers. HNPCC is an autosomal dominant genetic disorder that is caused by an inherited germline mutation in a DNA mismatch repair (MMR) gene [3].

The mismatch repair system consists of several nuclear proteins that are responsible for maintaining genetic stability by repairing base-to-base mismatches and insertion/deletion loops that arise during S phase. The inactivation of this system causes genomic instability and a predisposition to cancer [4]. Therefore, colon cancers from LS patients often exhibit microsatellite instability [5]. Mutations in four genes are primarily responsible for LS: MLH1, MSH2, MSH6, and PMS2.

Seventy percent of HNPCC families identified on the basis of family history criteria have a germline mutation in an MMR gene. About 80% of these MMR mutations are found in the MLH1 and MSH2 genes, 10% in MSH6, and < 5% in PMS2 [6]. The majority of germline MMR DNA mutations lead to a truncated protein product.

One problem with identifying LS is that often the diagnosis occurs only after the affected individual develops cancer. Another issue with detecting LS is that the currently available tests for detecting DNA MMR protein abnormalities are based on DNA sequencing, an expensive, time consuming process available mainly at commercial laboratories. To address this problem, we considered the development of a practical immunoassay based on the theoretical consideration that protein expression follows gene dosage. We previously showed [7] that *immortalized *lymphocytes from LS patients have a reduced level of their corresponding full length MMR protein, either MLH1 or MSH2. In the current study we determined whether MSH2 and MLH1 proteins can also be detected in *fresh *lymphocytes, which would make any population based assay more practical. Showing that one can determine the levels of MLH1 and MSH2 in lymphocytes from fresh blood samples could be the basis for developing a population-based screening method that more accurately detects LS trait carriers before they develop cancer. To establish proof of principle for this assay, we analyzed fresh blood samples from a population of individuals who are at high risk for having a germline MMR mutation

## Methods

### Materials

Human colorectal cancer cell lines (SW480, LoVo, HCT116), culture media (RPMI-1640, MEM, F-12 K), Fetal Bovine Serum (FBS), Trypsin/EDTA and antibiotics were purchased from American Type Culture Collection (ATCC). Antibodies were from the commercial sources indicated (Table 1). M-PER mammalian protein extraction reagent was from Pierce Biotechnology. Anti-mouse-IgG-HRP conjugated detection antibody, protease inhibitor cocktail, PMSF, 2-mercaptoethanol, PHA, penicillin, and streptomycin were from Sigma-Aldrich. Lymphoprep was from Axis-Shield. Human IL-2 was a gift from Dr. Martin Cannon, University of Arkansas for Medical Sciences, Little Rock, AR.

**Table 1:** Commercially available monoclonal and polyclonal antibodies used for detection of MLH1 and MSH2 proteins on western blots.

**No**.	Names	Catalog Number	Company
**Monoclonal Antibodies**			
1	Anti-MSH2(Ab-2) mouse mAb(FE11)	NA27	EMD Calbiochem, Gibbstown, NJ
2	MLH1	554073	BD Pharmingen, San Diego, CA
3	Anti-MSH2(Ab-1)mouse mAb(GB12)	NA26T	Calbiochem, San Diego, CA
4	Anti-MLH1(Ab-1)mouse mAb(14)	NA28	Calbiochem, San Diego, CA
5	MLH1	Sc-56159	Santa Cruz, Santa Cruz, CA
6	MLH1	Sc-56161	Santa Cruz, Santa Cruz, CA
7	MSH2	Sc-56163	Santa Cruz, Santa Cruz, CA
8	MSH2	556349	BD Pharmingen, San Diego, CA
**Polyclonal Antibodies**			
1	MLH1(N-20)	Sc-581	Santa Cruz, Santa Cruz, CA
2	MSH2 (N-20)	Sc-494	Santa Cruz, Santa Cruz, CA
3	Anti-MSH2 (Ab-3)	Pc57	Calbiochem, San Diego, CA
4	Anti-MLH1 (Ab-2)	Pc56	Calbiochem, San Diego, CA
5	Rabbit anti-MSH2	A300-020A	Bethyl Labs, Montgomery, TX
6	MLH1	2549.00.02	Sdix, Newark, DE

### Isolation of Lymphocytes

After IRB approval and signed informed consent, venous blood was collected from patients using EDTA-containing vacutainer tubes. Samples were collected from individuals undergoing genetic counseling for hereditary colon cancer in the Familial Cancer Clinic at the Helen F Graham Cancer Center, Christiana Care Health System (Newark DE). Samples were de-identified and processed within 24 hours to isolate lymphocytes. Lymphocytes were separated by density gradient centrifugation using Lymphoprep. Briefly, blood samples were diluted 2-fold with PBS, pH 7.4. An aliquot of 20 ml diluted blood was layered over 15 ml of Lymphoprep in 50 ml Falcon centrifuge tubes and centrifuged at 1000 g for 20 min at room temperature in a Sorvall RC 6 Plus centrifuge using an SH 3000 swinging bucket rotor. Lymphocytes were harvested from the buffy coat; monocytes from the plasma layer. Lymphocytes were diluted 3-fold with PBS (pH 7.4), washed by centrifugation at 350 g, and washed twice more at 300 g (5 min each), at room temperature. Lymphocytes were counted by Trypan blue staining and cultured (1 × 10^6 ^cells/ml RPMI-1640 medium). The lymphocyte yield was ~1 × 10^6 ^cells per ml of blood.

### Cell Culture

Lymphocytes were cultured in RPMI-1640 medium supplemented with 10% FBS, 1% penicillin/streptomycin, 5 mM 2-mercaptoethanol and 10 ul/ml human-IL-2 at 37°C in a 5% CO_2 _atmosphere. Immortalized lymphocytes were grown in the same medium as fresh lymphocytes but without 2-mercaptoethanol and human-IL-2. Human colon cancer cell lines (SW480, LoVo, HCT116) were cultured and maintained using established procedures (ATCC).

### Stimulation with PHA

To enhance the expression of MMR proteins, lymphocytes were stimulated with a mitogen, PHA. Cell lysates were then prepared. For optimized expression of MLH1 and MSH2 proteins, fresh blood lymphocytes were routinely stimulated with 10 ug PHA for 48 hrs.

### Western blotting

Cell lysates were prepared in M-PER Mammalian protein extraction reagent containing protease inhibitor cocktail and following the manufacturer's instructions. Protein concentrations were determined by colorimetry [8]. Western blotting was done as described previously [9]. For simultaneous detection of MLH1 and MSH2, a combination of anti-hMSH2 (Ab-2) and hMLH1 monoclonal antibodies from Calbiochem and BD Pharmingen, respectively, were used at 1:1000 dilution in the same western blot.

### Densitometry Analysis

Density of the bands of interest on a western blot was determined by scanning of the x-ray film and highlighting the band area using a BioRad Gel 2000 documentation system and its software. The actual density of each band was the value obtained after subtracting the background taken from the same x-ray film with an equivalent area. Ratios between MLH1 and MSH2 were used to compare variations among patient samples. The smaller of the two values, MLH1 or MSH2, always became the numerator; the larger became the denominator. Thus, the smaller the ratio is relative to 1.0, the greater the decrease of the protein in the numerator with respect to the level of protein in the denominator.

## Results

To develop an immunoassay that is accurate, we screened a number of commercially available monoclonal and polyclonal antibodies (Table 1) using western blotting to detect full-length MLH1 and MSH2 proteins in cell lysates from established colorectal carcinoma cell lines. The results for polyclonal antibodies were inconsistent. Most polyclonal antibodies did not show sufficient specificity to be used for measuring MLH1 and MSH2 levels. Those that did work did not produce consistent results; thus, we were unable to use them for quantitative detection of these proteins (data not shown). However, we found that two of the monoclonal antibodies (No. 1 and 2 in Table 1) can quantitatively detect full-length MLH1 and MSH2 proteins and which could be combined in a multiplex fashion to detect both proteins in a single assay.

**Figure 1:**
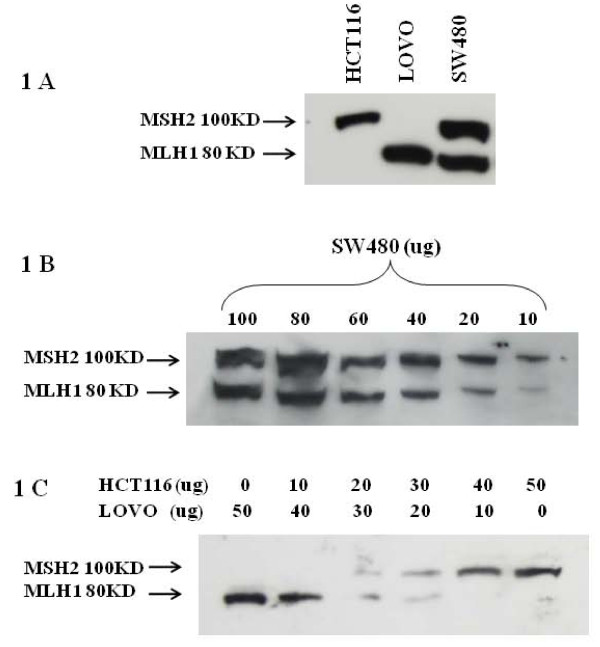
**Detection of MLH1 and MSH2 proteins using combined MLH1 and MSH2 monoclonal antibodies on the same blot**. **(A) **HCT116 and LoVo cells were used as controls for the absence and presence of MLH1 and MSH2 proteins, respectively, whereas SW480 cells were used for the presence of both these proteins. There was no apparent cross-reactivity. **(B) **Different concentrations of SW480 cell extracts were used for western blotting to establish simultaneous detection of both proteins. Results indicated that the combined antibodies were able to specifically detect their respective antigens in a dose dependent manner. MLH1 and MSH2 proteins could be detected in samples containing as little as 10 ug of total cell protein. **(C) **Detection of MLH1 and MSH2 proteins on western blots with a mixture of varying amounts of HCT116 and LoVo cell lysates. Results show that the combinations of these two monoclonal antibodies were able to detect MLH1 and MSH2 proteins even when these proteins were present in a sample in different proportions.

Figure 1A shows that both hMLH1 (80 kDa) and hMSH2 (100 kDa) proteins were detected on the same blot using a mixture of monoclonal anti-MLH1 and anti-MSH2 antibodies (mAbs) that specifically detect one or the other of these proteins. Colorectal adenocarcinoma cell lines - SW480, HCT116 and LoVo - were used as positive controls. SW480 expresses both full length MLH1 and MSH2; HCT116 expresses only full length MSH2; LoVo expresses only full length MLH1. These antibodies detected these proteins in a concentration dependent manner in dilution experiments using SW480 cells that contain both MLH1 and MSH2; the limit of detection was 10 ug of total cellular protein (Figure 1B). These antibodies also detected these proteins in a concentration dependent manner using a mixture of LoVo and HCT116 cell lysates when the lysates from these cell lines were mixed together in varying proportions (Figure 1C).

**Figure 2:**
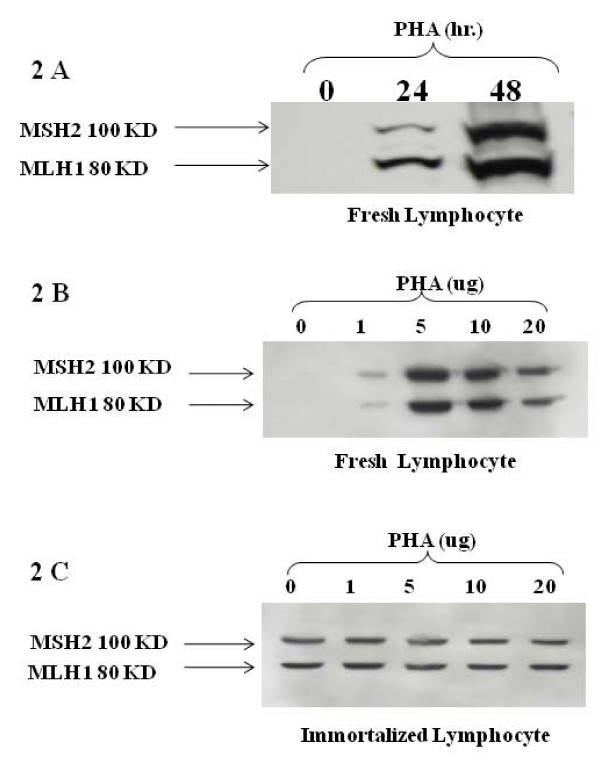
**Expression of MLH1 and MSH2 proteins in fresh blood and in immortalized lymphocytes following PHA stimulation**. **(A) **Time-dependent stimulation of MLH1 and MSH2 proteins in fresh blood lymphocytes following PHA treatment. The expression of MLH1 and MSH2 proteins increased in a time dependent manner. These proteins were often not detectable without PHA stimulation. **(B) **Dose response of fresh lymphocytes to PHA. Lymphocytes were stimulated with the indicated concentrations of PHA for 48 hrs. The expression of MLH1 and MSH2 proteins in fresh blood lymphocytes increased in a dose-dependent manner. **(C) **Dose response of immortalized lymphocytes to PHA. There was no effect of PHA on immortalized lymphocytes. MLH1 and MSH2 proteins were detectable even without PHA stimulation.

To detect these MMR proteins and determine their ratio in lymphocytes from fresh human blood samples, we isolated lymphocytes and treated them under the conditions described in Materials and Methods. Baseline levels of MLH1 and MSH2 protein were often not detectable in fresh lymphocytes using western blot assays. However, when these lymphocytes were cultured with phytohemagglutinin (PHA), a mitogen, the expression of MLH1 and MSH2 increased in a dose- and time-dependent manner, making levels of these MMR proteins readily detectable in fresh lymphocytes (Figure 2A). MLH1 and MSH2 levels increased equally after stimulation by PHA (Figure 2B). MLH1 and MSH2 were readily detectable in immortalized lymphocytes and PHA treatment did not affect the expression of these proteins (Figure 2C). Moreover, PHA treatment of isolated, fresh monocytes did not enhance MSH2 and MLH1 expression.

**Figure 3:**
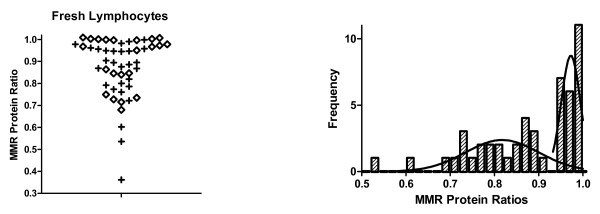
**DNA mismatch repair protein ratios for fresh lymphocyte samples from a population of individuals that were at high risk for having a germline MMR mutation**. The left panel shows a scatter plot of MMR ratios. The "+" signs represent ratios where MLH1 was less than MLH2. The diamonds represent ratios where MSH2 was less than MLH1. Because these plots were largely superimposable, we pooled them to establish the histogram shown in the right panel. The histogram shows that there is a bimodal distribution of MMR ratios. Moreover, the proportion of cases in the smaller mode (left most curve in right panel) is ~28%, which is very close to the proportion of patients (25%) at our recruitment site that have historically proved to have a germline MMR mutation.

Analysis of fresh lymphocytes (PHA treated) from a cohort of patients (N > 50 subjects) at *high risk *for LS, showed a bimodal distribution of MMR ratios (see histogram in Figure 3). The ratios ranged from 0.3 to 1.0 and peaks (mean ± SDE) were at 0.97 ± 0.02 and 0.81 ± 0.08. Stratification of results (shown as a scatter plot in Figure 3) shows that the MLH1 protein level is substantially reduced ("plus" symbols) in some fresh lymphocyte samples and MSH2 is reduced ("diamond" symbols) in other samples. In contrast, analysis of PHA stimulated fresh lymphocytes from *normal controls *revealed an MMR ratio close to 1.0 (Table 2). Analysis of normal controls and SW480 cells shows that the assay is highly reproducible (overall mean ± SDE = 0.96 ± 0.03). A bimodal distribution was not seen for normal healthy control subjects.

**Table 2:** Reproducibility of the Western Blotting Assay*

Cells	Mean ± SDE
SW480	0.989 **± **0.006
WBC Control 1	0.980 **± **0.018
WBC Control 2	0.967 **± **0.031
WBC Control 3	0.954 **± **0.059
WBC Control 4	0.921 **± **0.074

* Mean and standard deviation from MMR protein ratios determined from three different experiments on fresh WBCs from 4 control cases as well as SW480 colon cancer cells used as an internal control.

## Discussion

A main finding of this study is that levels of MMR proteins can readily be measured in lymphocytes from fresh blood samples if the lymphocytes are first stimulated to proliferate by PHA. This supports our idea that a practical immunoassay for MMR proteins can be developed and used to screen for patients affected with the LS trait before they develop cancer. These findings are consistent with results from our previous study [7] in which we assayed immortalized lymphocytes.

Our assay using two monoclonal antibodies appears to be specific because it accurately detects MLH1 and MSH2 in control cell lines that contain one or the other or both of these proteins (Figure 1A) and the assay also detects MLH1 and MSH2 proteins in mixing experiments where these proteins are present in varying proportions (Figure 1C). Our immunoassay also appears to be sensitive since it will detect MLH1 and MSH2 proteins in a sample from SW480 cells that contains as little as 10 ug of cellular protein (Figure 1B). Moreover, our assay appears to have an acceptable level of precision in that it is highly reproducible (Table 2).

The fact that MLH1 and MSH2 are not readily detected in untreated fresh lymphocytes or monocytes is likely due to the fact that they are not rapidly proliferating. This is supported by the fact that MLH1 and MSH2 are detectable in immortalized lymphocytes [7], which are proliferative cells by virtue of the fact that they have been transfected with an attenuated Epstein Barr Virus (EBV) and PHA treatment has little affect on MLH1 and MSH2 levels in these already proliferative cells. It should be noted that colon cancer cell lines (e.g., SW480) are also proliferating cells and have readily detectable levels of MMR proteins. The importance of our finding that PHA stimulation makes MLH1 and MSH2 detectable in fresh lymphocytes has relevance to the development of a practical immunoassay for the identification of carriers of an LS trait in a population-based setting.

A second finding is that the distribution of MMR ratios among individuals in a genetic counseling program, which includes carriers of an LS trait, was bimodal (Figure 3) with one peak close to 1.0 (less likely to be affected) and another lower than 1.0 (more likely to be affected). A bimodal distribution was not seen for healthy controls. This suggests that a subpopulation within the cohort of individuals at high risk for LS has substantially reduced levels of one of the two MMR proteins, which is what we predicted. This finding is consistent with our previous retrospective study [7] that also found a bimodal distribution. That earlier study was done using immortalized lymphocytes and involved individuals with a known MMR genotype, those who carried the LS trait and those who did not.

Our findings are consistent with other studies [10,11] that report microsatellite instability (MSI) in lymphocytes from LS patients - including ones with germline MSH2 or MLH1 mutations. If lymphocytes from LS patients have MSI, it can be assumed that they have reduced levels of the wild type DNA mismatch repair protein caused by the corresponding germline mutation.

Another study by Marra et al [12] reported that MSH2 protein levels are decreased in immortalized lymphocytes from LS patients carrying known MSH2 germline mutations. They claimed that MLH1 protein levels were not similarly decreased in immortalized from patients carrying known MLH1 germline mutations. In their study, MSH2 and MLH1 levels were normalized relative to beta-tubulin levels and the level of MMR proteins in the heterozygous immortalized lymphocyte extracts was reported as percentage of the mean value of three controls. Their quantification of MMR protein levels was not determined as a ratio between MSH2 and MLH1 as we did in our study. While they claim that MLH1 protein levels were not decreased in MLH1^+/- ^cells, their reported data for MLH1 levels show a wide range of variation. Their calculated mean MLH1 protein level for the 12 MLH1^+/- ^lymphocyte cell lines was 86.8% of controls, but the range was 44% to 117% of controls with the standard deviation (SDE) being ± 19.1. Given that there was such a wide range, it seems as though MLH1 levels are actually reduced in several of their immortalized lymphocyte lines that are heterozygous for MLH1 mutations.

Although our immunoassay is based on protein expression, it should have several advantages over assays based on genetic tests. Genetic tests such as DNA sequencing and microsatellite analysis are accurate, but are more expensive, take longer to do, and are mainly available at commercial laboratories. Also, DNA sequencing and microsatellite analysis is often done on patients who already have cancer and have a positive history of cancer. For these reasons, using genetic tests is not a practical way to screen large populations. In contrast, an immunoassay such as ours could be advanced to an automated diagnostic platform that is inexpensive, rapid and widely available. Moreover, since an immunoassay does not detect a genetic alteration, testing should not require a signed informed consent, which would be required for patients undergoing genetic testing. Indeed, in testing tumor tissues from patients who have already developed colon cancer for LS, an immunoassay (i.e., immunohistochemistry) is often used as a pre-screen before gene sequencing. In this case, immunohistochemistry is considered to be more feasible than the more complex strategy of genotyping for MSI [13]. Moreover, immunohistochemistry on tumor tissue is widely available, cost effective, and widely done without informed consent. This illustrates that clinicians are quite familiar with the use of immunoassays to diagnose human diseases.

Also, we are currently in the process of advancing our immunoassay to a sandwich ELISA format, which should have enhanced sensitivity, and would be a step closer to a commercially available clinical assay. Finally, this study bears repeating as a prospective study in which genotyping is done, which was beyond the scope of our current pilot study.

## List of Abbreviations

LS: Lynch Syndrome; HNPCC: hereditary non-polyposis colorectal cancer; MMR: DNA mismatch repair; PHA: phytohaemagglutinin; FBS: fetal bovine serum; MEM: Minimum Essential Medium; PMSF: p-amidinophenyl methanesulfonyl fluoride; IL-2; interleukine-2; SDE: standard deviation.

## Competing interests

The authors declare that they have no competing interests.

## Authors' contributions

SH performed experiments, analyzed data and participated in writing; BMB, NA, AAA and JZF conceived the idea, designed and supervised the study, and participated in data analysis and writing of the manuscript; MP, CS and ZJA provided genetic counseling. All authors read and approved the final manuscript.
